# Gender Separation Increases Somatic Growth in Females but Does Not Affect Lifespan in *Nothobranchius furzeri*

**DOI:** 10.1371/journal.pone.0011958

**Published:** 2010-08-03

**Authors:** Michael Graf, Alessandro Cellerino, Christoph Englert

**Affiliations:** 1 Molecular Genetics, Leibniz Institute for Age Research, Fritz Lipmann Institute (FLI), Jena, Germany; 2 Biology of Ageing, Leibniz Institute for Age Research, Fritz Lipmann Institute (FLI), Jena, Germany; Lund University, Sweden

## Abstract

According to life history theory, physiological and ecological traits and parameters influence an individual's life history and thus, ultimately, its lifespan. Mating and reproduction are costly activities, and in a variety of model organisms, a negative correlation of longevity and reproductive effort has been demonstrated. We are employing the annual killifish *Nothobranchius furzeri* as a vertebrate model for ageing. *N. furzeri* is the vertebrate displaying the shortest known lifespan in captivity with particular strains living only three to four months under optimal laboratory conditions. The animals show explosive growth, early sexual maturation and age-dependent physiological and behavioural decline. Here, we have used *N. furzeri* to investigate a potential reproduction-longevity trade-off in both sexes by means of gender separation. Though female reproductive effort and offspring investment were significantly reduced after separation, as investigated by analysis of clutch size, eggs in the ovaries and ovary mass, the energetic surplus was not reallocated towards somatic maintenance. In fact, a significant extension of lifespan could not be observed in either sex. This is despite the fact that separated females, but not males, grew significantly larger and heavier than the respective controls. Therefore, it remains elusive whether lifespan of an annual species evolved in periodically vanishing habitats can be prolonged on the cost of reproduction at all.

## Introduction

Among the many theories of ageing, life history theory combines evolutionary aspects with ecological and physiological traits. Life histories, and thus fitness, are shaped by these traits, which usually negatively correlate and, hence, are referred to as trade-offs [1], [2], [3], [4], [5]. One among the most studied trade-offs concerns the costs of reproduction on lifespan and ageing. Costs of reproduction denotes the investment to reproduce at the expense of future reproductive potential by either increased mortality or reduced fecundity [6], [7]. These costs are thought to either emerge from limited resources that have to be allocated between costly processes in terms of trade-offs [8] or being an immediate consequence of reproduction itself, that inflicts direct somatic damage [9].

A number of experiments in various models such as *Caenorhabditis elegans* [10], *Drosophila melanogaster* [6], *Musca domestica* [11] and *Callosobruchus maculatus* [12], [13], demonstrate an inverse correlation between reproduction and longevity. Likewise, longevity and low fecundity were found to be associated in birds and mammals [14], [15]. Conservation of this fecundity-longevity trade-off from invertebrates up to vertebrates speaks for an evolutionary preserved concept. However, Grandison et al. [16] very recently demonstrated that high fecundity and longevity are not mutually exclusive and can actually be uncoupled in *D. melanogaster* by methionine supplementation during caloric restriction. This influence of nutrition on fecundity and lifespan provides a possible explanation for a number of publications reporting contradicting findings on the cost of reproduction in other species. Reinhard and Köhler [17] did not find a cost of reproduction in the meadow grasshopper *Chorthippus*. In parallel it was shown in the Mediterranean fruit fly *Ceratitis capitata* that mating increased short-term mortality, however, in the long term mortality was decreased [18]. After all, more recent studies in captive populations of birds and mammals further demonstrated that reproductive investment and longevity do not correlate [19] and that ageing can even be delayed by reproduction as shown in mole rats [20]. In captive bred fish, analysis up to now has mainly focused on the impact of reproductive effort on growth [21], [22], while to our knowledge an analysis with respect to the possible trade-off between costs of reproduction and lifespan under laboratory conditions has not yet been reported.

In order to be able to perform short-term longevity studies in a vertebrate, we have begun to establish the extremely short-lived killifish *Nothobranchius furzeri* (*N. furzeri*) as a new model for ageing research [23]. The annual fish *N. furzeri* belongs to the teleosts and is found in seasonal ponds throughout South-Eastern Africa. The embryos endure the dry period in desiccation resistant eggs, which are arrested in a diapause state. With onset of the rainy season, progression of development is induced, eventually leading to hatching of the embryos. *N. furzeri* displays rapid growth and sexual maturation, since ponds dry up within few weeks. Interestingly, Valdesalici and Cellerino showed that this extraordinary short lifespan is maintained under laboratory conditions [23]. It could be further shown that *N. furzeri's* maximum lifespan in captivity correlates with duration of the rainy season in the respective sampling area, ranging from 12 weeks in semi-arid areas up to 45 weeks in sub-humid regions [24]. Under these conditions expression of ageing dependent markers (e.g. accumulation of lipofuscin, increasing neurofibrillation, decrease in locomotive activity and learning) was found to increase upon ageing of *N. furzeri*. When subjected to caloric restriction, resveratrol treatment as well as lowering of ambient water temperature, the expression rate of these markers was delayed while lifespan was prolonged [24], [25], [26], [27], [28].

To address the potential trade-off between longevity and reproduction in *N. furzeri* we have analyzed the impact of gender separation on lifespan. Given *N. furzeri's* peculiar life history, in particular its short period for mating and reproduction, we expect *N. furzeri* to invest substantial amounts of its energetic resources in production of offspring. Hence, repression of reproduction should significantly influence *N. furzeri's* life history, thus leading to a considerable surplus of energetic resources. However, although reproductive effort (clutch size per female) was greatly reduced, yet not abolished in separated females, a significant extension of lifespan could not be observed in either sex. This is despite the fact that separated females but not males, grew significantly larger and heavier than the respective controls. These data suggest that in an annual species that is adapted to ephemeral habitats a trade-off between the costs of reproduction and lifespan does not exist.

## Results

In the present work, we wanted to address the impact of mating and reproduction on lifespan in the short-lived killifish *Nothobranchius furzeri*. Most studies of *N. furzeri* have used the GRZ strain, which is extremely short-lived but highly inbred [24], [29]. As inbreeding depression is likely to affect fecundity, we decided to use the recently collected wild-derived strain MZM-04/10. This strain is genetically heterogeneous and was described elsewhere [24].

When the animals had reached sexual maturity (5 weeks), they were separated into three experimental groups: (a) males-only, (b) females-only and (c) mixed sexes. Subsequently, growth and lifespan of all animals was recorded. Data presented in this paper are based on two experimental trials, each carried out with an independent batch of eggs. As we did not observe differences in lifespan of the individual groups between trials, we compiled the data in order to enhance statistical power.

### Lifespan and demographic analysis

**Figure 1:**
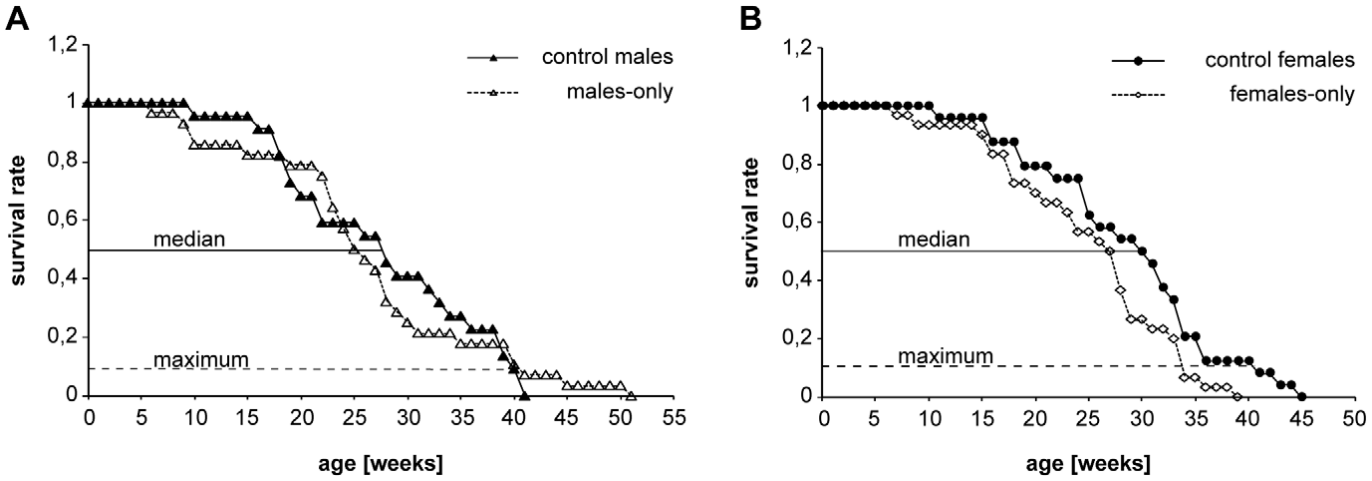
Survival rates for males and females of the *Nothobranchius furzeri* strain MZM-04/10. Animals were maintained in 40 l tanks as males-only (A) or females-only groups (B) and in mixed-sex control groups. The graphs represent the lifespan of control males (n = 22), males-only (n = 28), control females (n = 24) and females-only (n = 30) recorded during 2 independent trials. Median (50% survival) and maximum lifespan (10% survival) is indicated.

As shown in figure 1A males-only (n = 28) and control males (n = 22) showed median lifespans of 25 weeks and 28 weeks, respectively; 10% survival was reached at 40 weeks for both groups. Log rank analysis did not detect significant differences in age-related mortality in the two groups (χ^2^ = 0.001902, df = 1, p = 0.9652).

Females-only (n = 30) showed decreased median (27 vs. 30 weeks) and maximum lifespan (37 vs. 41 weeks) when compared with control females (n = 24) (1B). These differences did not reach significance in a Log rank test (χ^2^ = 3.216, df = 1, p = 0.0729). A specific test for 10% survivorship did not indicate significance either (Fisher's exact test, p = 0.31). In order to estimate sample size required for statistical significance of a 10% difference at 50% and 10% survival, we performed power analysis. Calculating for two independent groups (Fisher's exact test, two-tailed, α = 0.05, power 1−β = 0.8) a total sample size of 186 animals was computed. As this is the sample size required at 50% or 10% survival, initial total sample size would have to be 372 and 1860 animals, respectively. Given the demanding housing and breeding conditions for *Nothobranchius* these numbers are beyond the capacity of a research lab. Thus, on the basis of our data we conclude that separation of the sexes has no major influence on lifespan in *N. furzeri*.

### Development of body size and weight

**Figure 2:**
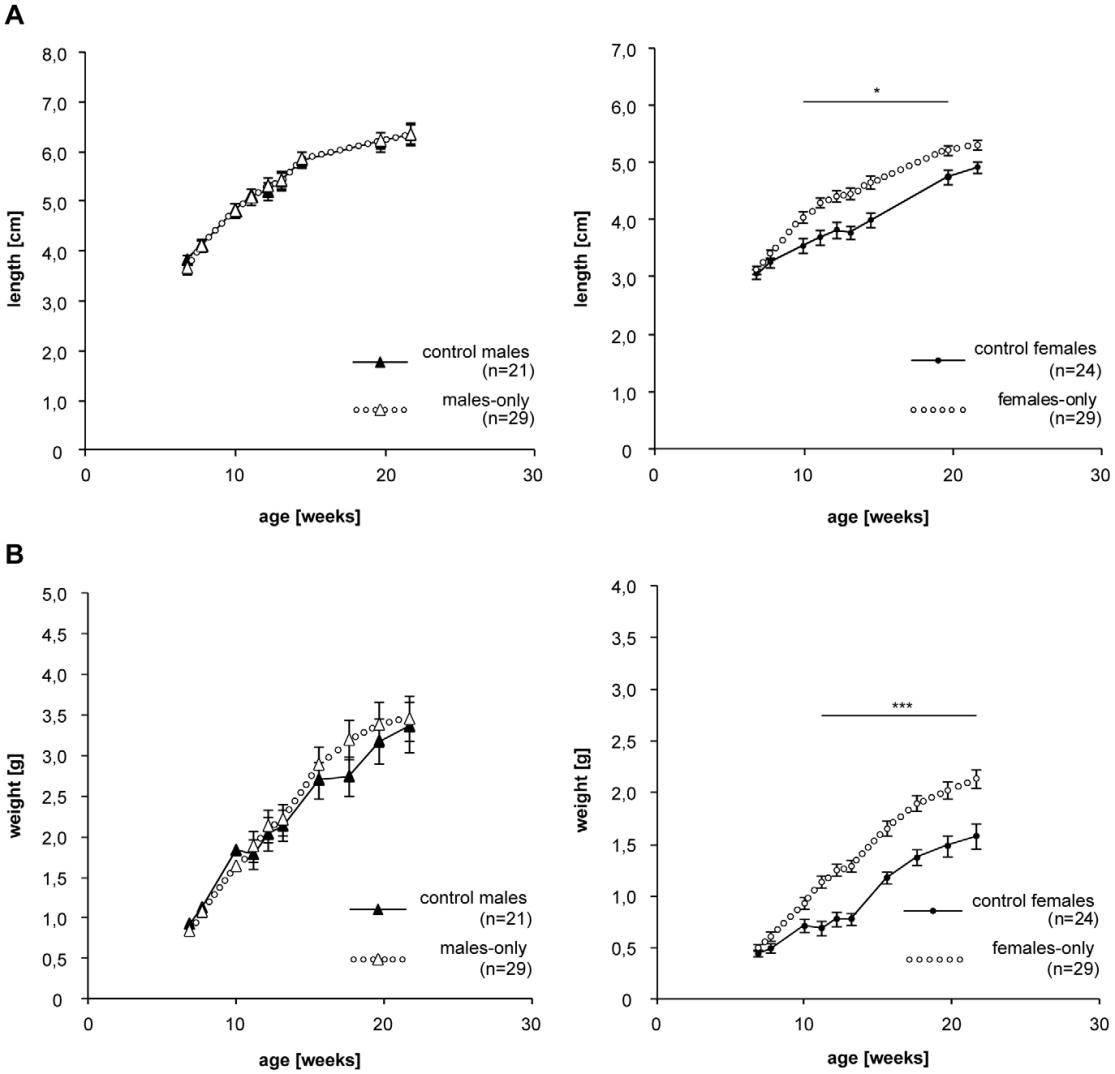
Age dependent changes in mean body weight and size in gender separated and control animals. Determination of length (A) and body weight (B) of males-only and control males (left) and females-only and control females (right). Graphs show mean ± sem. Significance for given data points were calculated by Bonferroni post hoc test.

Body length and weight were monitored as additional parameters. No statistically significant difference in body length or body weight was observed between controls and males-only at any age (fig. 2A, B). In contrast, females-only grew significantly larger and heavier than their reproducing siblings (two-way ANOVA: p<0.0001). At the age of 10 weeks, mean body length of females-only was 12% increased compared to the controls (fig. 2A, Bonferroni post hoc test: p<0.01). This difference increased until the age of 13 weeks to 15.3% (Bonferroni post hoc test: p<0.001) and remained significant until the age of 20 weeks (9.2%; Bonferroni post hoc test: p<0.05). The body weight was increased as well, ranging from 25–39% (fig. 2B, p<0.001). As animals in the tanks were not tagged, we could not record individual life history traits and test for correlation between size and longevity. Given that it would further not be possible to separate the effects on growth from preferential mortality of animals in defined size classes, body size measurements were finished before the experimental groups reached 50% survival.

### Production and release of eggs

**Figure 3:**
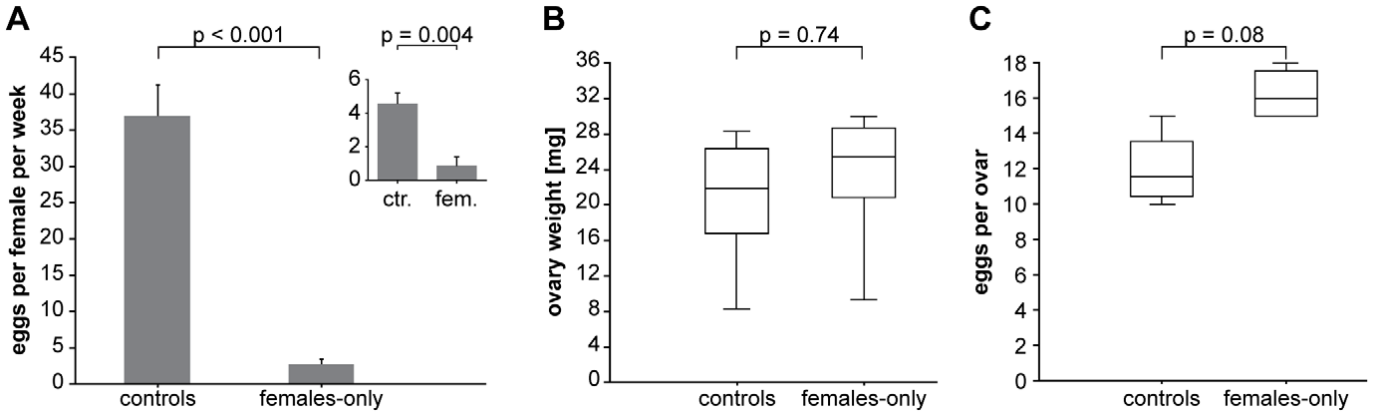
Analysis of reproductive effort. (A) Eggs laid into spawning boxes per female per week were recorded in a female-only (n = 12) and a control female cohort (n = 6) over 5 weeks beginning at the age of 5 weeks. Presented as inset are eggs laid per female per week when the tank bottom was 100% covered with breeding substrate. (B) At the age of 14 weeks ovaries of 4 females from each group were dissected and weighed. In both groups animals were observed with one ovary of smaller size (controls: 1, females-only: 2). These were included in the analysis. (C) Number of eggs in the ovary was counted in both groups (number of ovaries = 4). For this analysis only normal sized ovaries were used. Box plots show median, interquartile range and total range by lines, boxes and whiskers, respectively.

To estimate the reduction in reproductive investment and to address whether the significant increase in weight of separated females might be caused by the retention of eggs, we performed additional experiments. We first kept 12 females separated after the age of 5 weeks and monitored egg laying on a daily basis for 5 weeks, providing spawning boxes according to our standard procedures. These boxes covered nearly half of the total bottom area. The number of laid eggs per week in females-only was drastically reduced when compared to the controls (fig. 3A, controls: 36.9±4.3, separated: 2.7±0.8, mean ± sem, p<0.001). As separated females still released eggs without male stimuli, cannibalism of eggs released outside the spawning substrate had to be considered. Hence, a second trial was set up with sand covering 100% of the bottom area. Here, we still observed a significant 5-fold decrease in mean egg release upon gender separation (controls: 4.5±1.2, separated: 0.9±1, mean eggs per female per week ± sem, p = 0.0036). It is noteworthy that egg laying activity was generally reduced in the second experiment, most likely due to seasonal conditions. However, in both setups we find a decrease in egg release upon gender separation. In order to address whether also less eggs were produced in females-only, animals were sacrificed at the age of 14 weeks and the mass of the ovaries was determined. Also, mature eggs present in the gonads were counted. As presented in figure 3 there was no significant difference in ovary mass (fig. 3B, controls: 20.9±2.4 mg, separated: 25.7±2.4 mg, mean ± sem, n = 8 per group, p = 0.74) as well as number of eggs retained (fig. 3C, controls: 12±1.1, separated: 16.3±0.8, mean ± sem, n = 4 per group, p = 0.08). These data indicate a reduction in reproductive efforts in gender-separated females. The data further suggest that while separated females show a significant decline in egg laying the increased body mass of those animals when compared to the mixed controls cannot be explained by storage of eggs.

## Discussion

Our data on lifespan and growth of *Nothobranchius furzeri* upon gender separation shed first light on trade-offs in this new ageing model.

Animals used for this study were derived from two subsequent batches of eggs, thus reducing possible epigenetic effects as well as genotype by environment influences on survival data and growth curves. In both male and female cohorts our study does not reveal a statistically significant impact of repressed reproduction on lifespan. Although we cannot further distinguish between costs of mating and costs of egg and sperm production in this setup, it is striking that upon reducing these combined costs, somatic maintenance and thereby longevity is not increased in *N. furzeri*. Given the short existence of *N. furzeri's* ephemeral habitat, one could speculate that extending lifespan by increasing investment into somatic maintenance and longevity will not result in higher offspring numbers and thus not increase Darwinian fitness.

We further show that separated females display an at least 5-fold decrease in egg release, most likely due to the lack of male key stimuli. Although a slight but non-significant increase in eggs retained in the ovaries of separated females was found, the overall number of eggs that were produced was far below the number of those in females of the mixed cohort. This was associated with a significant increase of somatic growth. The underlying mechanisms remain unclear. Egg retention and ovary hyperplasia cannot account for this effect since ovary mass was only slightly increased in separated females. We would speculate that repression of reproduction in *N. furzeri* affects the somatotrophic axis, which in addition to regulating growth is involved in the regulation of fecundity and via the insulin/insulin-like growth factor 1 pathway also in the regulation of lifespan [30]. The engagement of gonadal steroids in the regulation of the somatotrophic axis has been reported for ovariectomized rats [31]. Whether repressed egg production indeed leads to reduced steroid release of the gonads of *N. furzeri* has yet to be investigated.

It is intriguing that the character of the trade-off between reproduction and somatic growth is apparently sex-specific. An obvious explanation would be that the investment into germ cell production is sex-specific. Generally, egg production is considered to be more costly than sperm production, since vast resources are spent on ovary activity and synthesis of yolk precursors. In birds an increase between 16–27% regarding resting metabolic rate during egg production has been reported [32].

Gender separation does not only obviate reproduction, but also alters social structure. It directly reduces social stress for females through elimination of male harassment. In contrast to the situation for males, we did not observe female against female aggressions and female hierarchies have not been reported for *Nothobranchius* species. In contrast, because of steady rivalry among males intrasexual social stress is more pronounced in males. As reviewed by Neumann [33] *Nothobranchius sp.* is not rigorously territorial but males have a strict dominant hierarchy associated with bright and intense coloration of only few big and strong animals when kept in groups. Rivaling males display extensive impressing behaviour followed by physical attacks, eventually culminating in jaw biting until one animal surrenders. Hence, male intrasexual competition is costly and would not be expected to decrease significantly upon gender separation. Thus, disregarding the costs of germ cell production and considering only social stress, one would predict that females should save more energy upon gender separation than males.

In summary, we demonstrated that gender separation in *Nothobranchius furzeri* sex-specifically influences somatic growth in females but not lifespan. This might at least in part be related to *N. furzeri's* unique life cycle but also suggests that resources saved by decreasing reproductive effort are not necessarily invested in longevity. To test the effect of the repression of reproduction more directly, gonad ablation experiments could be used in the future. It will also be important to identify the cause of death of the individual animals of *N. furzeri* so that it will be possible to assess whether the differences in physiology and behaviour of the sexes also result in a distinct repertoire of ageing-associated pathologies.

## Materials and Methods

### Fish strains and animal husbandry

The laboratory strain MZM-04/10 was collected during a field trip in Mozambique in 2004 and has been described [24]. Adult animals were kept in 40 l tanks at 26°C water temperature in a 12 h dark-light cycle and fed with frozen red mosquito larvae (Chironomidae) twice a day. Water was constantly filtered by air-driven foam filters and tanks were cleaned twice a week by aspirating off debris and changing 50% of the water. For mating and breeding sand-filled boxes were provided at the bottom of the tank, wherein eggs could be laid. Those were collected twice a week. Fertilized eggs were incubated on wet peat moss and embryonic development was observed by visual inspection. Upon completion of embryonic development, i.e. when a golden glimmer of the retina could be observed hatching was induced. For this water was added to the embryos together with peat moss and incubated over night at 26°C. Upon hatching embryos were transferred into 40 l tanks and fed with freshly prepared brine shrimp (*Artemia*) until they reached approximately 1.5 cm in length. Subsequently, food was changed to red mosquito larvae. Further details on the maintenance of *Nothobranchius sp.* are referred by Genade et al. [34].

### Experimental groups

For this study two subsequent trials were carried out, using two independent hatches. Three trial groups, namely males-only, females-only and mixed-sex groups were set up when the animals had reached 5 weeks of age and showed first signs of sex dimorphism. Initial animal densities in these trials were 13–16 animals per 40 l tank. Tanks were checked twice a day and dead animals were instantly recorded and removed. Based on these data survival was calculated on a weekly basis. During the trial we did not adjust population density caused by declining group size. To exclude effects arising from competition for food, ample food was provided and widely distributed over the tank in order to avoid fighting at the site of feeding. In addition, tanks and water systems were completely separated, thus preventing animals from sensing any hormones or compounds from other tanks. Tanks were also separated physically so that animals from one tank could not see animals from another tank.

All experiments were conducted following the “Principles of laboratory animal care” and the current version of the German Law on the Protection of Animals. A license for holding and breeding of the animals including organ preparation has been granted by the local authorities (# 2684-05-04-FLI-Jena-01/06).

### Measurements

Body mass was assessed by weighing individual animals on a laboratory scale. For the analysis of body size, animals were placed in a glass bottom bowl on millimetre paper and photographed from top using a digital camera. Digital pictures were analysed using the Adobe Photoshop pixel measuring tool. During early development, these evaluations were performed once a week. From age 13 weeks onward data were recorded once every 2 weeks.

To quantify egg release one additional control group (n = 6 females + 6 males) and female-only group (n = 12) was set up. Both groups were housed in 40 l tanks (tank bottom: 49×32 cm) with spawning boxes provided (2 boxes each 15×22.5 cm) covering approximately half the bottom area. Eggs were collected and counted daily over 5 weeks beginning at the age of 5 weeks. Based on these data an index for the number of eggs laid per week per female was calculated. At the age of 14 weeks ovaries from 4 females of each group were dissected and weighed. Four ovaries per group were further analysed for egg content. Here, mature eggs undergoing hydration were counted. During dissection individuals each with one ovary of clearly smaller size were observed in both groups (controls: 1, females-only: 2). Those were included in the measurement of ovary mass but not in analysis of egg content.

Cannibalism of eggs outside the spawning boxes was addressed by an additional experiment. One control group (n = 6 females+6 males) and one female-only group (n = 12) were set up in tanks with sand covering 100% of tank bottom area. For four weeks eggs were collected by sieving the substrate.

### Statistical Analysis

Statistical analysis were performed using GraphPad Prism version 5.03 for windows (GraphPad Software, San Diego California USA, www.graphpad.com) Survival curves of gender separated and control cohorts were compared by Log rank analysis. Statistical significance at 10% survival was tested by Fisher's exact test. One-way ANOVA was used to test differences in ovary mass and number of eggs. Differences in body growth and size were tested for statistical significance by two-way ANOVA followed by Bonferroni post hoc test. Power analysis was performed using the freeware tool GPower [35].
